# Docosahexaenoic acid attenuates neuropathological alterations in a rodent model of neonatal bilirubin-induced encephalopathy

**DOI:** 10.3389/fmed.2026.1633947

**Published:** 2026-04-02

**Authors:** Yi Yao, Wenhong Zhang, Yunqian Chi, Zihan Zhang, Yunhe Wang, Wei Hao

**Affiliations:** 1 Clinical Medicine, Basic Medical College of Guangxi Medical University, Nanning, Guangxi, China; 2 Department of Pediatric, Shandong Provincial Hospital Affiliated to Shandong First Medical University, Jinan, Shandong, China

**Keywords:** bilirubin-induced encephalopathy, CtBP1, docosahexaenoic acid, ferroptosis, miR-155-5p

## Abstract

**Background:**

Neonatal bilirubin encephalopathy (kernicterus) results from toxic accumulation of unconjugated bilirubin in the brain, often leading to irreversible neurological damage. Docosahexaenoic acid (DHA), an omega-3 fatty acid with anti-inflammatory and antioxidant properties, may attenuate bilirubin-induced neuropathological alterations in neonates.

**Objectives:**

This study evaluated whether DHA mitigates neuropathological and molecular changes in a rodent model of bilirubin-induced encephalopathy via the CTBP1/miR-155-5p/KDM5A pathway and oxidative stress regulation.

**Methods:**

In a cross-sectional experimental study, 48 neonatal Sprague-Dawley rats (7 days old) were allocated to Control, bilirubin encephalopathy (BE) and BE + DHA groups (*n* = 16 each). Bilirubin (120 mg/kg i.p.) was administered to induce encephalopathy. DHA (100 mg/kg by gavage) was given daily for 3 days. Physical status, neuro-behavior (righting reflex, negative geotaxis), biochemical markers (serum bilirubin, neuron-specific enolase [NSE], oxidative stress indices), and histopathology were assessed. Gene and protein expression of CTBP1, KDM5A and miR-155-5p were measured (RT-qPCR, Western blot).

**Results:**

DHA improved neurobehavioral scores and was associated with a faster decline in serum bilirubin levels during the later observation period. Serum bilirubin peaked at ∼15 μmol/L at 24 h in BE and declined to 10 μmol/L by 72 h, whereas DHA accelerated clearance to ∼5 μmol/L at 72 h (control ∼5 μmol/L). Brain bilirubin and NSE were significantly elevated in BE and mitigated by DHA (each ∼60% reduction vs. BE, *p* < 0.01). DHA also reduced cortical apoptosis (TUNEL-positive cells ∼10% vs. 25% in BE, *p* < 0.01). Molecular analysis showed bilirubin-induced upregulation of miR-155-5p (∼3-fold) with downregulation of CTBP1 and KDM5A (∼50–60% of control); DHA partially restored CTBP1/KDM5A levels and lowered miR-155-5p (∼1.4-fold, *p* < 0.01 vs. BE). Markers of oxidative stress and ferroptosis were elevated in BE (e.g., malondialdehyde + 175%, *p* < 0.001) and blunted by DHA (+ 50% vs. control, *p* < 0.01).

**Conclusion:**

DHA administration significantly attenuated bilirubin-induced neurotoxicity in neonatal rats. Neuroprotection by DHA was associated with changes in the CTBP1/miR-155-5p/KDM5A axis and reduced oxidative stress–related markers.

## Introduction

Bilirubin encephalopathy (BE), also known as kernicterus, is the unifying term encompassing the spectrum of bilirubin-related neurologic injury (1, 2). BE manifests as encephalopathy with motor deficits, hearing loss, and cerebral palsy in survivors, reflecting irreversible injury to the basal ganglia and brainstem nuclei if not promptly treated (3, 4). Due to the neonatal brain’s high susceptibility to bilirubin toxicity, effective therapeutic approaches are required not only to reduce bilirubin levels but also to protect and repair neural tissues damaged by bilirubin (5).

Currently, the standard interventions for severe neonatal hyperbilirubinemia are phototherapy and exchange transfusion. While these treatments reduce circulating bilirubin, they do not always avert bilirubin neurotoxicity in extreme cases or underlying cellular injury (6). Thus, there is an impetus to explore adjunct neuroprotective strategies that can mitigate the neuropathological consequences of bilirubin encephalopathy. Recent advances in neonatal neurocritical care have drawn attention to nutritional and pharmacological neuroprotectants that go beyond bilirubin reduction alone (7, 8). One such agent is DHA, a long-chain omega-3 polyunsaturated fatty acid integral to brain structure and function. DHA is a major constituent of neuronal membranes and is essential for proper neurodevelopment and synaptogenesis. It also possesses anti-inflammatory and antioxidant properties that could counteract bilirubin-induced neurotoxicity (8–10).

Early preclinical studies have shown that DHA supplementation exerts neuroprotective effects in neonatal brain injury models. Specifically, in models of neonatal hypoxia–ischemia and traumatic brain injury, DHA improved functional and histological outcomes by reducing neuroinflammation, oxidative damage, and apoptosis (9, 10). However, to our knowledge, no comprehensive study has yet evaluated DHA in an animal model of bilirubin encephalopathy. The present study therefore represents the first detailed investigation of DHA’s effects on neurobehavioral, biochemical, and molecular outcomes in neonatal rats with bilirubin encephalopathy, focusing on the CTBP1/miR-155-5p/KDM5A pathway and ferroptosis-related mechanisms (10).

A regulatory pathway of interest in this context is the C-terminal binding protein 1 (CTBP1)/microRNA-155-5p (miR-155-5p)/lysine (K)-specific demethylase 5A (KDM5A) signaling axis. CTBP1 functions as a transcriptional co-regulator involved in gene repression and neuronal development. MiR-155-5p is a pro-inflammatory microRNA that regulates immune signaling and neuronal injury responses in the central nervous system. KDM5A is a histone H3K4 demethylase that participates in epigenetic regulation of gene expression and cellular stress responses (11, 12).

Bilirubin toxicity may disrupt this delicate balance—for instance, unconjugated bilirubin has been shown to increase miR-155-5p expression in neural cells, potentially silencing KDM5A and impairing cellular stress responses. By modulating this CTBP1/miR-155/KDM5A pathway, DHA might restore homeostatic gene regulation and enhance the brain’s resilience to bilirubin (13). Another critical mechanism in bilirubin-induced neurotoxicity is ferroptosis, a form of regulated cell death driven by iron-dependent lipid peroxidation. Excess bilirubin can catalyze oxidative membrane damage and may perturb iron homeostasis in the developing brain. Ferroptosis is characterized by depletion of glutathione peroxidase 4 (GPX4) activity and accumulation of lipid peroxides; consistent biomarkers include elevated prostaglandin-endoperoxide synthase-2 (PTGS2/COX-2) and acyl-CoA synthetase long-chain 4 (ACSL4) (14).

Neuronal ferroptosis has been implicated in acute brain injuries and neurodegeneration. We posit that bilirubin neurotoxicity may involve ferroptotic processes, and that DHA’s antioxidant action could interrupt this form of cell death. DHA can intercalate into neuronal membranes and is a precursor to neuroprotectin D1, which upregulates antioxidant enzymes and suppresses lipid peroxidation (15). In addition, DHA might preserve levels of GPX4 or activate Nrf2-dependent antioxidant responses, thereby preventing the execution of ferroptosis. Indeed, related studies demonstrate that enhancing antioxidant defenses (e.g., via Nrf2/HO-1 activation or by compounds like Orexin-A) can alleviate ferroptotic damage in brain injury models (16).

Recent syntheses have mapped the experimental landscape of bilirubin encephalopathy (BE) models, including hemolytic and bilirubin-injection rodent models, and have reviewed potential neuroprotective strategies beyond bilirubin-lowering therapies (17). Emerging proteomic and mechanistic studies suggest that ferroptosis contributes to BE-related brain injury, highlighting lipid-peroxidation pathways involving ACSL4 and GPX4 as potential therapeutic targets. In experimental rat models, curcumin has been reported to reverse cerebellar hypoplasia and improve behavioral abnormalities through anti-inflammatory and antioxidant mechanisms. These findings indicate that targeting oxidative stress and regulated cell death pathways may represent a promising therapeutic strategy for bilirubin-induced neurotoxicity.

A previous study by our group reported clinical and biochemical outcomes of DHA supplementation in neonatal hyperbilirubinemia. However, that work primarily focused on therapeutic effects in a clinical context and did not investigate the molecular mechanisms underlying DHA-mediated neuroprotection. The present study extends this work by employing a controlled neonatal rat model of bilirubin encephalopathy to investigate the involvement of the CTBP1/miR-155-5p/KDM5A signaling pathway and ferroptosis-related oxidative stress in DHA-mediated neuroprotection.

## Materials and methods

### Study design and animals

This cross-sectional experimental study was conducted from March 2024 to March 2025. A total of 48 healthy neonatal Sprague-Dawley rats (7 days old, both sexes) were obtained from the institutional breeding colony. Pups were randomly assigned to one of three groups (16 pups per group): Control, BE and BE + DHA. Each litter was distributed across groups to minimize confounders.

### Induction of bilirubin encephalopathy

We employed a neonatal bilirubin encephalopathy (BE) model based on intraperitoneal administration of unconjugated bilirubin in postnatal day 7 (PND7) Sprague–Dawley rat pups. This experimental paradigm produces acute bilirubin neurotoxicity by directly elevating circulating unconjugated bilirubin levels and has been widely used to reproduce the neuropathological features of kernicterus in neonatal rodents. In contrast to hemolytic models induced by agents such as phenylhydrazine (PHZ), which generate hyperbilirubinemia through erythrocyte destruction, the bilirubin injection model allows controlled elevation of bilirubin concentrations and precise timing of neurotoxic exposure. Therefore, it is particularly suitable for investigating the mechanisms of bilirubin-induced neuronal injury and evaluating potential neuroprotective interventions (8, 14).

### DHA Treatment

DHA (100 mg/kg/day, oral gavage) was administered on PND7, PND8 and PND9, beginning ∼1 h after bilirubin injection on PND7. Behavioral testing on PND9 and PND11 was performed before daily gavage to avoid acute effects. Controls and BE group pups received equivalent vehicle gavage (8).

### Physical and neurobehavioral assessments

Neurobehavioral assessments were conducted to evaluate sensorimotor development and neurological function in neonatal rats. Righting reflex testing was performed on PND9, whereas negative geotaxis testing was performed on PND11, after completion of the DHA treatment period.

#### Righting reflex (PND9)

Each pup was placed in the supine position on a flat surface, and the time required to turn over onto all four paws was recorded. Three trials were performed for each animal with at least 5 min of rest between trials, and the average latency was calculated.

#### Negative geotaxis (PND11)

Each pup was placed head-downward on a 30° inclined board, and the time required to rotate 180° to face upward was recorded. Three trials were performed per pup, and the median latency was used for analysis. Animals unable to complete the task within 30 s were assigned a maximum score of 30 s. The negative geotaxis test is widely used to assess vestibular-motor coordination and neurological development in neonatal rodents (15).

#### Neurological scoring system

Neurological status was evaluated using a modified bilirubin encephalopathy scoring system adapted for neonatal rodents. Animals were assessed for characteristic neurological signs associated with bilirubin neurotoxicity, including lethargy, abnormal posture (opisthotonus), impaired suckling or feeding behavior, and reduced spontaneous movement. Each parameter was scored on a 0–2 scale (0 = normal, 1 = mild abnormality, 2 = severe abnormality). The total neurological score therefore ranged from 0 to 8, with higher scores indicating more severe neurological dysfunction. Neurological assessments were performed by investigators blinded to group allocation.

### Biochemical measurements

**FIGURE 1:**
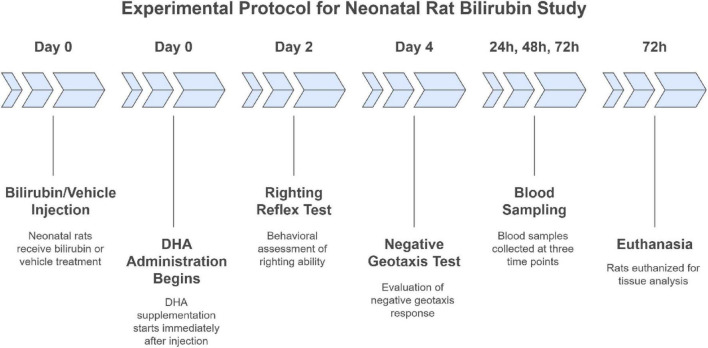
Experimental timeline and sample allocation in the neonatal rat model of bilirubin encephalopathy.

The experimental timeline and group allocation are summarized in Figure 1. Additional methodological details are provided in Supplementary Figure 1. Bilirubin injection was performed on postnatal day 7 (PND7), which was defined as time 0. Subsequent measurements at 24 h, 48 h, and 72 h therefore corresponded to PND8, PND9, and PND10, respectively. DHA treatment was administered daily from PND7 to PND9. Behavioral testing was conducted on PND9 (righting reflex) and PND11 (negative geotaxis). Blood samples for bilirubin measurement were collected at 24, 48, and 72 h after bilirubin injection, and subsets of animals were euthanized at 72 h (PND10) for biochemical, molecular, and histopathological analyses (Figure 1).

Brain tissue bilirubin content was quantified by a solvent extraction method: cerebrum samples were weighed, homogenized, and mixed with acetone to extract bilirubin, which was then measured via an automatic biochemical analyzer at 450 nm. Results were expressed as micrograms of bilirubin per gram of brain tissue (μg/g). Oxidative stress markers were evaluated in brain cortex homogenates collected at 72 h. Malondialdehyde (MDA), an end-product of lipid peroxidation, was measured by thiobarbituric acid reactive substances assay (using a commercial kit, Sigma) and normalized to protein content (nmol MDA per mg protein). We also measured reactive oxygen species (ROS) levels in cortical cells using a 2′,7′-dichlorofluorescein diacetate (DCFDA) fluorescence assay. Briefly, fresh cortex tissue was minced and incubated with DCFDA; the fluorescence intensity (excitation 485 nm, emission 535 nm) was recorded on a microplate reader, reflecting ROS accumulation. In addition, we examined expression of key proteins involved in oxidative stress and ferroptosis pathways, including GPX4, PTGS2 (COX-2), and ACSL4, by Western blot.

Experimental timeline and group allocation of neonatal rat model of BE. On postnatal day 7 (PND7), BE and BE + DHA pups received intraperitoneal bilirubin (120 mg/kg); controls received vehicle. DHA was administered orally on PND7–PND9. Righting reflex and negative geotaxis testing were performed on postnatal days PND9 and PND11, respectively. DHA treatment was administered from PND7 to PND9; therefore, the PND11 behavioral assessment was conducted after completion of the treatment period.

Serum bilirubin levels were quantified using a micro-volume spectrophotometric bilirubinometer calibrated for neonatal rat serum samples. This approach differs from the clinical biochemical analyzer used in our previous study, which was optimized for human samples. Brain tissue bilirubin levels were determined using an acetone-based solvent extraction method followed by spectrophotometric detection at 450 nm, allowing quantification of bilirubin deposition within neural tissue.

Neonatal Sprague–Dawley rats were randomly assigned to Control, BE, and BE + DHA groups (*n* = 16 per group). Bilirubin injection was performed on postnatal day 7 (PND7), defined as time 0. DHA was administered orally once daily from PND7 to PND9. Blood samples for serum bilirubin measurement were collected at 24 h (PND8), 48 h (PND9), and 72 h (PND10) after bilirubin injection. Behavioral testing was conducted on PND9 (righting reflex) and PND11 (negative geotaxis). At 72 h post-injection (PND10), subsets of animals were euthanized for biochemical, molecular, and histopathological analyses.

Neonatal Sprague–Dawley rats were randomly assigned to Control, BE, or BE + DHA groups (*n* = 16 per group). Bilirubin injection was performed on postnatal day 7 (PND7), defined as time 0. DHA was administered orally once daily from PND7 to PND9. Blood samples for serum bilirubin measurement were collected at 24 h (PND8), 48 h (PND9), and 72 h (PND10) after bilirubin injection. Behavioral testing was conducted on PND9 (righting reflex) and PND11 (negative geotaxis). At 72 h post-injection (PND10), subsets of animals were euthanized for biochemical, molecular, and histopathological analyses. The diagram illustrates the allocation of animals and timing of experimental procedures.

### Molecular analysis—*in vivo* (rat cortical tissue)

#### *In vivo* pathway expression (rat cortex)

##### CTBP1/miR-155-5p/KDM5A pathway expression in rat cortex

The expression levels of CTBP1, KDM5A, and miR-155-5p were evaluated in cortical tissue samples using quantitative reverse transcription polymerase chain reaction (RT-qPCR).

Total RNA was extracted from cerebral cortex tissue using TRIzol reagent (Invitrogen, United States) according to the manufacturer’s instructions. RNA concentration and purity were assessed spectrophotometrically. Complementary DNA (cDNA) synthesis was performed using the RevertAid First Strand cDNA Synthesis Kit (Thermo Fisher Scientific) for mRNA targets and the miScript II RT Kit (Qiagen, Germany) for microRNA analysis.

Quantitative PCR was carried out using SYBR Green Master Mix on an ABI Prism real-time PCR system. Gene expression levels of Ctbp1 and Kdm5a mRNA and miR-155-5p were determined using gene-specific primers. β-actin was used as the internal control for mRNA normalization, whereas U6 small nuclear RNA served as the reference control for miRNA quantification.

Relative expression levels were calculated using the 2−ΔΔCt method, with the Control group serving as the calibrator. All reactions were performed in triplicate to ensure reproducibility.

### *In Vitro* luciferase assay

To determine whether miR-155-5p directly targets Kdm5a, a luciferase reporter assay was performed in HEK293 cells. Cells were co-transfected with a pmirGLO reporter plasmid containing the Kdm5a 3′UTR, a miR-155-5p mimic or inhibitor, and/or a CTBP1 overexpression plasmid using Lipofectamine 3,000 (Thermo Fisher). After 48 h, firefly and Renilla luciferase activities were measured using the Dual-Luciferase Reporter Assay System (Promega). Firefly luciferase activity was normalized to Renilla luciferase activity.

### Gene expression (RT-qPCR)

At 72 h post-injection, rats (*n* = 6 per group) were anesthetized and decapitated for cortical tissue collection. Total RNA was extracted from cerebral cortex using TRIzol (Invitrogen, United States). cDNA synthesis was performed with the RevertAid kit (Thermo, United States) for mRNA and the miScript kit (Qiagen, Germany) for miRNA. Quantitative RT-qPCR was performed on an ABI Prism system using SYBR Green Mastermix. We quantified mRNA levels of Ctbp1 and Kdm5a, and miRNA levels of miR-155-5p normalized to U6 small RNA. Primers were designed based on rat sequences (miR-155-5p assay ID: rno-miR-155-5p). Relative expression was calculated using the 2^−ΔΔCt^ method, with the Control group as calibrator (fold change = 1.0).

### Protein expression (Western Blot)

Cortical tissue samples were lysed in RIPA buffer containing protease inhibitors. Protein concentrations were determined using the bicinchoninic acid (BCA) assay. Thirty micrograms (30 μg) of total protein per sample were separated by SDS–polyacrylamide gel electrophoresis and transferred onto polyvinylidene fluoride (PVDF) membranes.

Membranes were blocked with 5% non-fat milk in Tris-buffered saline with 0.1% Tween-20 (TBST) for 1 h at room temperature and then incubated overnight at 4°C with primary antibodies against CTBP1, KDM5A, GPX4, ACSL4, and COX-2 (PTGS2). After washing with TBST, membranes were incubated with horseradish peroxidase–conjugated secondary antibodies for 1 h at room temperature.

Protein bands were visualized using enhanced chemiluminescence and captured with a digital imaging system. Band intensities were quantified by densitometric analysis and normalized to β-actin, which served as the loading control.

### In *vitro* experiments—HEK293 luciferase reporter assay

To specifically test whether miR-155-5p directly targets Kdm5a and whether CTBP1 modulates this interaction, we performed a luciferase reporter assay in HEK293 cells (ATCC). This *in vitro* system was independent of the animal model and is therefore described separately. Cells were maintained in DMEM supplemented with 10% FBS. HEK293 cells were co-transfected with a pmirGLO reporter containing the 3′UTR of rat Kdm5a (with the predicted miR-155-5p binding site), a miR-155-5p mimic or inhibitor, and/or a CTBP1 overexpression plasmid using Lipofectamine 3,000 (Thermo Fisher). After 48 h, firefly and Renilla luciferase activities were measured using the Dual-Luciferase Reporter Assay System (Promega). The ratio of firefly/Renilla luciferase was normalized to control transfections to determine miR-155-5p–Kdm5a interactions.

### Tissue collection and regional selection

For biochemical assays with low-abundance markers (e.g., oxidative stress and ferroptosis indices), whole-brain homogenates were used to obtain sufficient tissue mass for accurate measurement. For histopathological, immunohistochemical, and Western blot analyses, cortical tissue was selected because it represents one of the primary targets of bilirubin neurotoxicity and showed the most pronounced neuropathological changes in our pilot work. In selected assays, the hippocampus and basal ganglia were also analyzed because these regions are known to be vulnerable to bilirubin toxicity and are clinically relevant to kernicterus (auditory and motor sequelae). This tiered approach allowed us to balance analytical sensitivity with region-specific vulnerability.

### Histopathological analysis

All histopathological analyses were conducted on brains collected at postnatal day 10 (PND10) to capture acute neuropathological changes before behavioral testing on PND11. For histology, at 72 h after bilirubin injection a subset of pups (*n* = 4 per group) was perfused transcardially with phosphate-buffered saline followed by 4% paraformaldehyde. Brains were post-fixed, embedded in paraffin, and coronal sections (5 μm) were cut at the level of the hippocampus and basal ganglia. Sections were stained with hematoxylin and eosin (H&E) following standard protocols.

An experienced neuropathologist blinded to group examined the sections under light microscopy. We qualitatively assessed neuronal morphology (pyknosis, necrosis), presence of bilirubin pigment (yellow discoloration in tissue), gliosis, and any hemorrhages. We also performed Nissl staining on adjacent sections to evaluate neuronal density in hippocampal CA regions and cortex. For apoptosis detection *in situ*, a TUNEL (terminal deoxynucleotidyl transferase dUTP nick-end labeling) assay was performed on some sections (ApopTag kit, Millipore). TUNEL-positive nuclei were counted in five random high-power fields (HPF) per section to quantify apoptotic cell percentage.

For histopathological quantification, three coronal sections per brain were analyzed. For each section, five random high-power fields (400 × magnification) were examined. Normal-appearing cortical neurons were defined as neurons with intact morphology, visible nuclei, and preserved Nissl substance. Neuronal vacuolation was assessed using a semi-quantitative scoring system (0–3) based on the proportion of neurons exhibiting cytoplasmic vacuolization. GFAP-positive astrocytes were scored using a 0–3 scale reflecting the intensity and distribution of astrocytic activation. All analyses were performed by a neuropathologist blinded to experimental group allocation.

To examine the temporal pattern of Cyclin D protein expression following bilirubin-induced injury, an additional independent cohort of neonatal rats was used for time-course immunohistochemical analysis. Animals were subjected to the same bilirubin injection protocol described above and were sacrificed at 1, 7, 14, and 28 days post-injury. Brain tissues were processed using the same fixation and sectioning procedures as described for the primary cohort. Immunohistochemical staining for Cyclin D1, Cyclin D2, and Cyclin D3 was performed on cortical sections. For each animal, three coronal sections were analyzed and five random high-power fields per section were evaluated under 400 × magnification. The number of Cyclin-positive cells was assessed semi-quantitatively by an investigator blinded to group allocation to evaluate the temporal progression of cell-cycle protein expression following bilirubin exposure.

### Statistical analysis

All data were tested for normality using the Shapiro–Wilk test and for homogeneity of variance using Levene’s test. Parametric data were analyzed using one-way ANOVA followed by Tukey’s *post-hoc* test. Non-parametric data were analyzed using Kruskal–Wallis tests followed by Dunn’s *post-hoc* test with Bonferroni correction. For time-course analyses (e.g., serum bilirubin levels over time), repeated-measures ANOVA (or mixed-effects models where appropriate) was employed to account for within-subject correlations. Statistical significance was set at *p* < 0.05 after correction for multiple comparisons.

### Ethical considerations

All animal procedures were approved by the Institutional Animal Care and Use Committee, and conducted in strict accordance with National Research Council guidelines for the care of laboratory animals. The study was also approved by the institutional research committee bearing Approval No. FMU/SHJ/2024/797.

## Results

### General clinical observations

Because the present study measured both serum and brain tissue bilirubin using different analytical methods than those used in our previous report, the absolute values and temporal kinetics are not directly comparable. However, the overall trend of bilirubin elevation following injection and subsequent reduction with DHA treatment remained consistent with the neuroprotective effects previously reported.

All bilirubin-injected rats developed visible jaundice within 12 h, confirming the induction of hyperbilirubinemia. Untreated BE rats appeared lethargic with reduced suckling and episodes of opisthotonic posturing by 48 h, indicative of acute bilirubin neurotoxicity. In contrast, BE rats treated with DHA maintained relatively normal feeding behavior and less frequent opisthotonus. No adverse effects of DHA were observed. Final body weights at day 11 were slightly lower in the BE group (mean 18.3 ± 1.2 g) compared to Control (19.5 ± 1.1 g, *p* < 0.05), whereas the DHA-treated group had intermediate weights (19.0 ± 1.3 g, not significant vs. Control).

### Neurological clinical evaluation

All bilirubin-injected rats developed visible jaundice within 12 h after bilirubin administration, confirming successful induction of hyperbilirubinemia. By 48 h post-injection, untreated BE rats displayed neurological signs consistent with acute bilirubin neurotoxicity, including lethargy, reduced suckling activity, and episodes of opisthotonic posturing.

**FIGURE 2:**
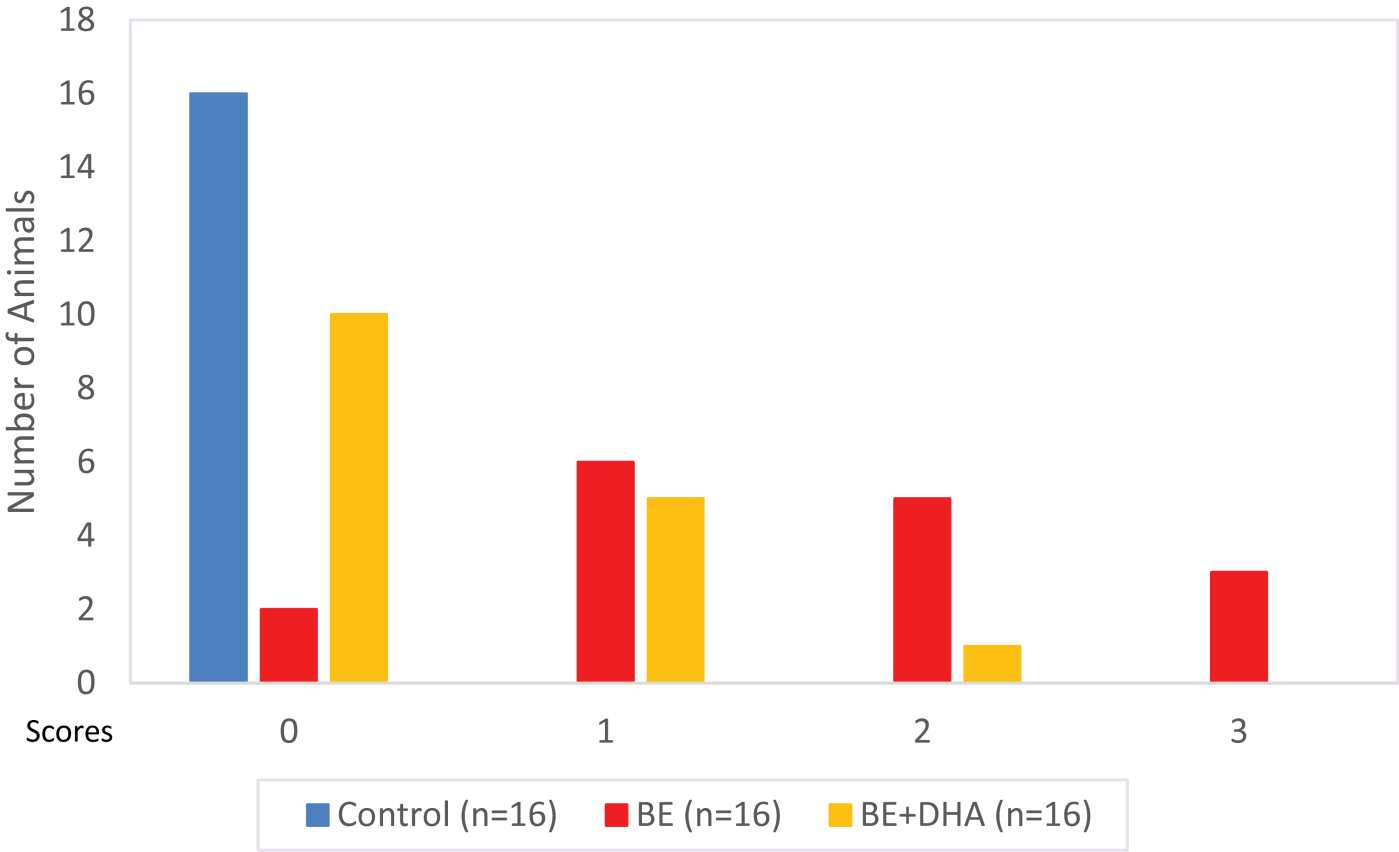
Frequency distribution of neurological scores in neonatal rats.

In contrast, rats in the BE + DHA group exhibited milder clinical manifestations, maintaining relatively normal feeding behavior and demonstrating fewer episodes of opisthotonus. These clinical observations corresponded with the quantitative neurological scoring results described below (Figure 2). No adverse effects related to DHA administration were observed.

Final body weights measured on postnatal day 11 (PND11) were slightly lower in the BE group (18.3 ± 1.2 g) compared with Control animals (19.5 ± 1.1 g, *p* < 0.05), whereas the DHA-treated group showed intermediate values (19.0 ± 1.3 g), which were not significantly different from controls.

To quantitatively evaluate neurological impairment, neurological status was assessed using the scoring system described in the Methods section. In the BE group, half of the animals (8/16, 50%) exhibited neurological scores ≥ 2, indicating moderate neurological impairment. In contrast, animals in the BE + DHA group predominantly displayed lower scores (0–1), consistent with milder neurological dysfunction and partial neuroprotection following DHA treatment (Figure 2).

Distribution of neurological scores among animals in each group at 72 h post-bilirubin injection. Although the scoring system allows a maximum value of 8, the observed scores in this experiment ranged from 0 to 3, indicating predominantly mild to moderate neurological impairment.

### Neurobehavioral tests

Neurobehavioral testing revealed significant sensorimotor impairment in untreated BE pups compared with controls.

For the righting reflex test (PND9), BE animals showed markedly prolonged response times (8.7 ± 2.1 s) relative to Control animals (3.2 ± 0.8 s, *p* < 0.01). DHA treatment significantly improved performance (4.5 ± 1.2 s, *p* < 0.05 vs. BE), approaching control levels.

Similarly, in the negative geotaxis test (PND11), BE pups required significantly more time to reorient on the inclined plane (15.4 ± 4.5 s) compared with Control animals (6.1 ± 1.9 s, *p* < 0.01). DHA treatment improved this response (8.0 ± 2.3 s, *p* < 0.05 vs. BE), indicating partial recovery of vestibular-motor coordination.

**FIGURE 3:**
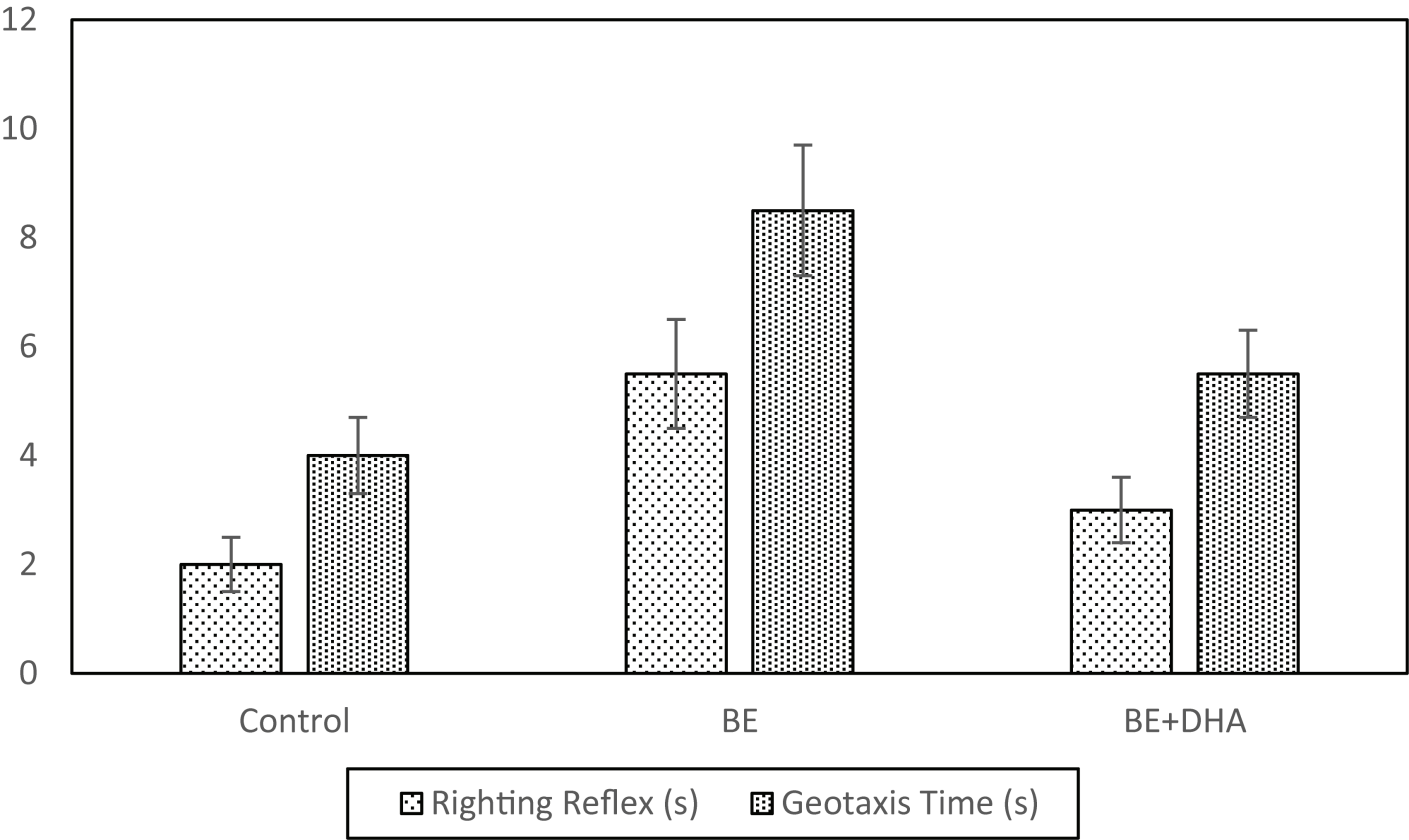
Neurobehavioral performance of neonatal rats on righting reflex and negative geotaxis tests.

These findings demonstrate that bilirubin exposure impaired neurobehavioral development, while DHA treatment significantly improved behavioral performance (Figure 3).

### Bilirubin levels in serum and brain tissue

**FIGURE 4:**
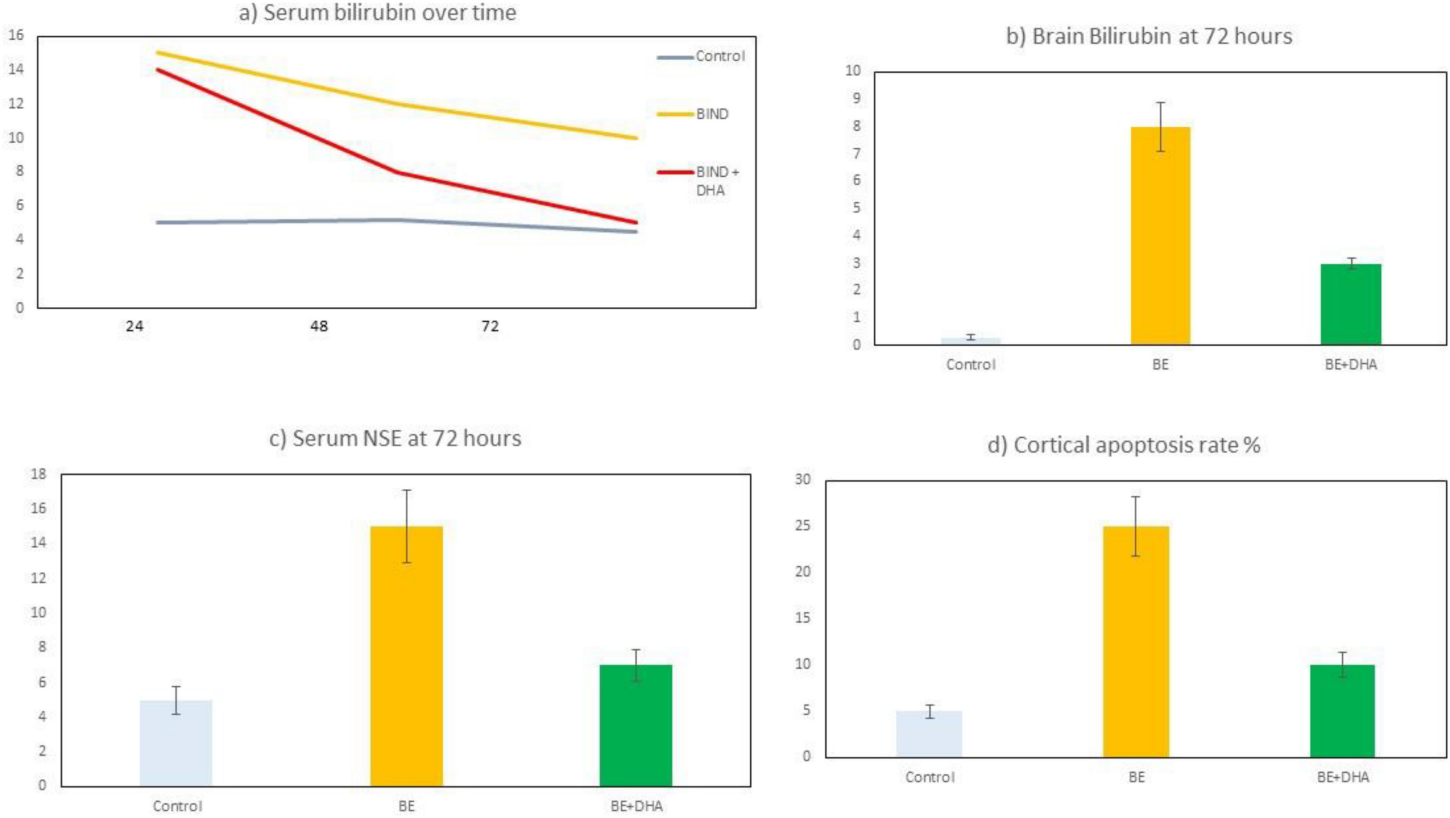
Bilirubin and neuronal injury markers in neonatal rats following bilirubin injection. **(a)** Serum bilirubin concentrations measured at 24, 48, and 72 h after bilirubin injection in Control, BE, and BE + DHA groups. **(b**) Brain bilirubin levels measured at 72 h post-injection. **(c)** Serum neuron-specific enolase (NSE) levels at 72 h, and **(d)** the percentage of TUNEL-positive apoptotic cells in cortical tissue. Data are expressed as mean ± SD. BE, bilirubin encephalopathy; DHA, docosahexaenoic acid.

Serum bilirubin levels were measured at 24, 48, and 72 h after bilirubin injection (Figure 4a and Table 1). Baseline bilirubin concentrations were similar across all groups prior to injection (approximately 5.0 μmol/L).

**TABLE 1:** Serum Bilirubin concentrations (μmol/L) at different time points after bilirubin injection.

Time (h)	Control	BE	BE + DHA
0	5.0	5.0	5.0
24	5.0	15.0	14.0
48	5.0	12.0	8.0
72	5.0	10.1	5.2

In the BE group, serum bilirubin increased markedly to 15.0 ± 1.3 μmol/L at 24 h, followed by a gradual decline to 12.0 ± 1.1 μmol/L at 48 h and 10.1 ± 1.0 μmol/L at 72 h. In contrast, the BE + DHA group showed a similar peak at 24 h (14.0 ± 1.5 μmol/L) but demonstrated a more rapid decline thereafter, reaching 8.0 ± 0.9 μmol/L at 48 h and 5.2 ± 0.8 μmol/L at 72 h.

By 72 h, serum bilirubin concentrations in the BE + DHA group were significantly lower than in the untreated BE group (*p* < 0.01).

Brain bilirubin accumulation measured at 72 h is shown in Figure 4b. Control animals exhibited minimal brain bilirubin levels (0.3 ± 0.1 μg/g tissue), whereas the BE group showed substantially elevated levels (8.0 ± 1.2 μg/g tissue). DHA treatment significantly reduced brain bilirubin accumulation to 3.1 ± 0.8 μg/g tissue (*p* < 0.01 vs. BE).

These findings indicate that bilirubin exposure resulted in marked increases in circulating and brain bilirubin levels, whereas DHA treatment was associated with lower bilirubin levels at later time points (Figure 4).

DHA-treated animals showed a faster decline in serum bilirubin levels over time compared with untreated BE animals.

While all groups started with similar baseline bilirubin levels (5.0 μmol/L), the BE group exhibited a sharp increase, peaking at 15.0 μmol/L at 24 h and gradually decreasing to 10.1 μmol/L by 72 h. In contrast, DHA-treated rats (BE + DHA) showed a faster reduction in bilirubin levels, declining to near-normal levels (5.2 μmol/L) by 72 h. This observation suggests that DHA may facilitate bilirubin clearance or redistribution in this experimental setting (Table 1).

### Neuronal injury biomarkers

Serum neuron-specific enolase (NSE) levels were measured at 72 h after bilirubin injection (Figure 4c and Table 2). NSE concentrations were significantly higher in the BE group (15.3 ± 2.4 ng/mL) compared with the Control group (5.2 ± 1.1 ng/mL, *p* < 0.001).

**TABLE 2:** Serum neuronal injury marker, brain tissue bilirubin levels, and oxidative stress indices in Control, BE, and BE + DHA groups at 72 h post-injection.

Group	NSE (ng/mL) (mean ± SD)	Brain Bilirubin (μg/g) (mean ± SD)	MDA (nmol/mg) (mean ± SD)
Control	5.3 ± 1.0	0.3 ± 0.1	2.0 ± 0.4
BE	15.2 ± 2.3	8.1 ± 1.2	5.6 ± 0.6
BE + DHA	7.3 ± 1.5	3.1 ± 0.7	3.0 ± 0.5

Values are presented as mean ± SD.

In the BE + DHA group, NSE levels were significantly lower than in the BE group (7.4 ± 1.5 ng/mL, *p* < 0.001) but remained slightly higher than Control values (*p* < 0.05).

Apoptotic cell death in cortical tissue was evaluated using TUNEL staining (Figure 4d). Control brains showed a low proportion of TUNEL-positive cells (∼5%). In contrast, the BE group exhibited a significantly higher percentage of apoptotic cells (25 ± 4%). DHA treatment significantly reduced the proportion of TUNEL-positive cells to 10 ± 3% (*p* < 0.01 vs. BE).

Immunohistochemical analysis of apoptosis-related proteins showed increased Bax and cleaved caspase-3 staining and reduced Bcl-2 expression in the BE group compared with Controls. In the BE + DHA group, Bax and cleaved caspase-3 staining were reduced, whereas Bcl-2 expression was increased relative to the BE group.

**FIGURE 5:**
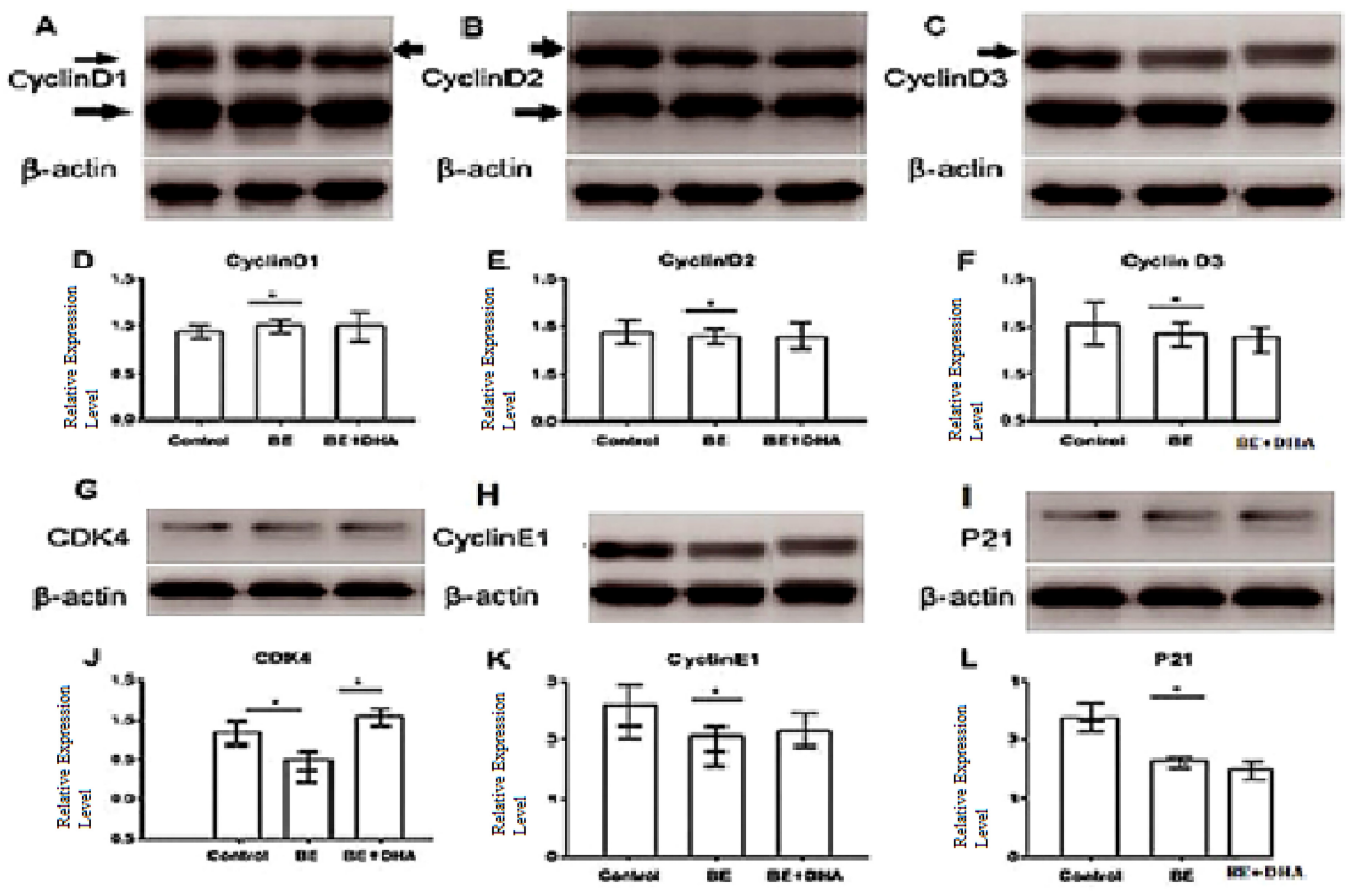
Expression of cell-cycle–related proteins in cortical tissue of neonatal rats. **(A–C)** Representative Western blot images showing the expression of Cyclin D1 **(A)**, Cyclin D2 **(B)**, and Cyclin D3 **(C)** in Control, BE, and BE + DHA groups. β-actin served as the loading control. **(D–F)** Densitometric quantification of Cyclin D1 **(D)**, Cyclin D2 **(E)**, and Cyclin D3 **(F)** protein expression normalized to β-actin. **(G–I)** Representative Western blot images showing the expression of CDK4 **(G)**, Cyclin E1 **(H)**, and P21 **(I)** in Control, BE, and BE + DHA groups. β-actin served as the loading control. **(J–L)** Densitometric quantification of CDK4 **(J)**, Cyclin E1 **(K)**, and P21 **(L)** protein expression normalized to β-actin. Data are presented as mean ± SD. Statistical comparisons were performed using one-way ANOVA followed by Tukey’s *post-hoc* test. *p < 0.05, *p < 0.01 vs. Control; #*p* < 0.05 vs. BE (observed in **J,L**).

Bilirubin-induced encephalopathy leads to dysregulation of key cell cycle proteins in the neonatal brain. Western blot analysis revealed significant upregulation of CyclinD1, CDK4, and CyclinE1 in the BE group compared to controls, indicating aberrant reactivation of the G1/S cell cycle transition in post-mitotic neurons, a process linked to apoptosis and neurodegeneration. Expression of cell-cycle–related markers differed significantly across groups (Figure 5). Compared with controls, BE cortex showed increased Cyclin D1, CDK4, and Cyclin E1 levels and reduced P21, suggesting aberrant cell-cycle re-entry. DHA treatment significantly reduced Cyclin D1, CDK4, and Cyclin E1 expression compared with the BE group; however, only CDK4 approached control levels, while Cyclin E1 remained moderately higher. P21 expression increased relative to BE but did not fully return to control values.

### Temporal immunohistochemical expression of cyclin d proteins

**FIGURE 6:**
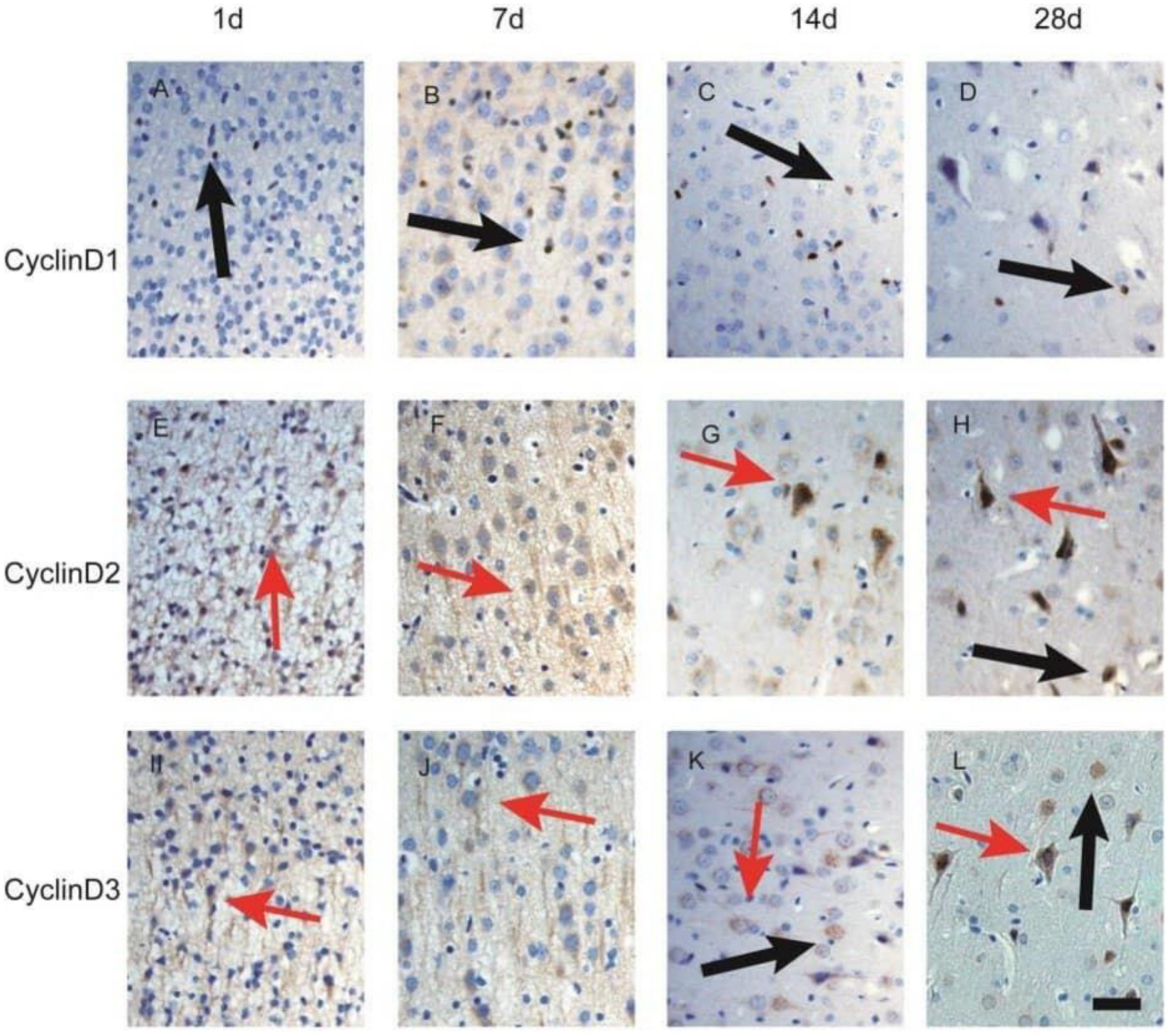
Temporal immunohistochemical expression of Cyclin D family proteins in cortical tissue following bilirubin exposure. Immunohistochemical staining for Cyclin D1 (**A–D)**, Cyclin D2 **(E–H)**, and Cyclin D3 **(I–L)** in cortical tissue at 1, 7, 14, and 28 days post-injury. Images represent tissue sections from animals exposed to bilirubin-induced encephalopathy. Arrows indicate Cyclin-positive cells. Scale bar = 50 μm.

To examine the temporal progression of Cyclin D family protein expression following bilirubin exposure, an independent cohort of neonatal rats was analyzed at multiple time points after injury (1, 7, 14, and 28 days post-injection) (Figure 6).

At 1 day post-injury, only a small number of Cyclin D–positive cells were observed in the cortex. The number and staining intensity of positive cells increased at 7 days, became more pronounced at 14 days, and remained elevated at 28 days, suggesting sustained activation of cell-cycle–related pathways during the subacute and chronic phases following bilirubin exposure.

### Histopathological findings at PND10

H&E-stained sections from BE pups at PND10 revealed classic kernicterus features: neuronal shrinkage, vacuolation and yellow pigment deposition. Quantitatively, the BE group showed a 32 ± 5% reduction in normal-appearing cortical neurons per high-power field compared to controls (*p* < 0.01). DHA treatment preserved cortical neuron counts (89 ± 6% of control, *p* < 0.05 vs. BE) and reduced vacuolation scores by ∼50%.

Nissl staining corroborated these findings: hippocampal CA1 neurons decreased by 28 ± 4% in BE rats, whereas DHA rats showed only a 10% decrease vs. control (*p* < 0.05).

TUNEL assay results (already partly described in your Figure 4d) showed 25 ± 4% apoptotic cortical cells in BE vs. 10 ± 3% in DHA-treated pups (*p* < 0.01).

Gliosis (GFAP immunoreactivity) was rated on a 0–3 scale: BE brains scored 2.5 ± 0.3 vs. 1.2 ± 0.2 in DHA-treated pups (*p* < 0.01).

Bilirubin pigment was detected in 65 ± 8% of BE cortical fields but in only 15 ± 5% of DHA-treated fields (*p* < 0.01).

### Oxidative stress and ferroptosis-related markers

Markers of oxidative stress were evaluated in cortical tissue at 72 h post-injection (Tables 2, 3).

**TABLE 3:** Oxidative stress and apoptosis markers.

Group	ROS (AU)	TUNEL-positive cells (%)
Control	100 ± 20	5 ± 1
BE	220 ± 30	25 ± 4
BE + DHA	120 ± 25	10 ± 3

Malondialdehyde (MDA), an indicator of lipid peroxidation, was significantly elevated in the BE group (5.6 ± 0.6 nmol/mg) compared with the Control group (2.0 ± 0.4 nmol/mg, *p* < 0.001) (Table 2). In the BE + DHA group, MDA levels were significantly lower than in the BE group (3.0 ± 0.5 nmol/mg, *p* < 0.01 vs. BE).

Reactive oxygen species (ROS) levels measured using a DCFDA fluorescence assay were also elevated in the BE group (220 ± 30 arbitrary units) compared with Control animals (100 ± 20 arbitrary units, *p* < 0.01) (Table 3). ROS levels in the BE + DHA group were lower than in the BE group (120 ± 25 arbitrary units, *p* < 0.01 vs. BE).

Expression of proteins associated with ferroptosis-related pathways, including glutathione peroxidase 4 (GPX4), prostaglandin-endoperoxide synthase 2 (PTGS2/COX-2), and acyl-CoA synthetase long-chain 4 (ACSL4), was analyzed by Western blot (Table 4). In the BE group, GPX4 expression was reduced (∼0.40 relative to β-actin), whereas COX-2/PTGS2 (∼3.0-fold) and ACSL4 (∼1.8-fold) were increased compared with Control animals. In the BE + DHA group, GPX4 expression was higher than in the BE group (∼0.85 relative expression), while COX-2/PTGS2 and ACSL4 levels were lower than in the BE group (∼1.2- and ∼1.1-fold, respectively) (Table 4).

**TABLE 4:** Ferroptosis-related protein expression (relative to β-Actin).

Protein	Control	BE	BE + DHA
GPX4	1.00	0.40	0.85
COX-2 (PTGS2)	1.00	3.00	1.20
ACSL4	1.00	1.80	1.10

The expression of ferroptosis-related proteins (GPX4, COX-2/PTGS2, and ACSL4) was evaluated by Western blot to assess the effects of bilirubin exposure and DHA treatment. In the BE group, ferroptotic activity is evident with a marked decrease in GPX4 (0.40) and increased expression of pro-ferroptotic markers COX-2 (3.00) and ACSL4 (1.80), compared to control levels. DHA treatment partially restored GPX4 expression (0.85) and reduced COX-2 and ACSL4 levels to near-control values (1.20 and 1.10, respectively), indicating that DHA suppresses ferroptosis and contributes to neural protection by stabilizing oxidative homeostasis (Table 4).

### CTBP1/miR-155-5p/KDM5A pathway expression *in vivo*

RT-qPCR analysis demonstrated significant dysregulation of the CTBP1/miR-155-5p/KDM5A pathway in cortical tissue following bilirubin exposure. Compared with the Control group, the BE group showed significant downregulation of CTBP1 (0.52 ± 0.10-fold) and KDM5A (0.60 ± 0.08-fold), whereas miR-155-5p expression increased to 3.0 ± 0.5-fold (*p* < 0.01).

In the BE + DHA group, miR-155-5p expression decreased to 1.4 ± 0.3-fold, while CTBP1 and KDM5A expression increased to 0.94 ± 0.15-fold and 1.12 ± 0.12 fold, respectively (*p* < 0.01 vs. BE).

**FIGURE 7:**
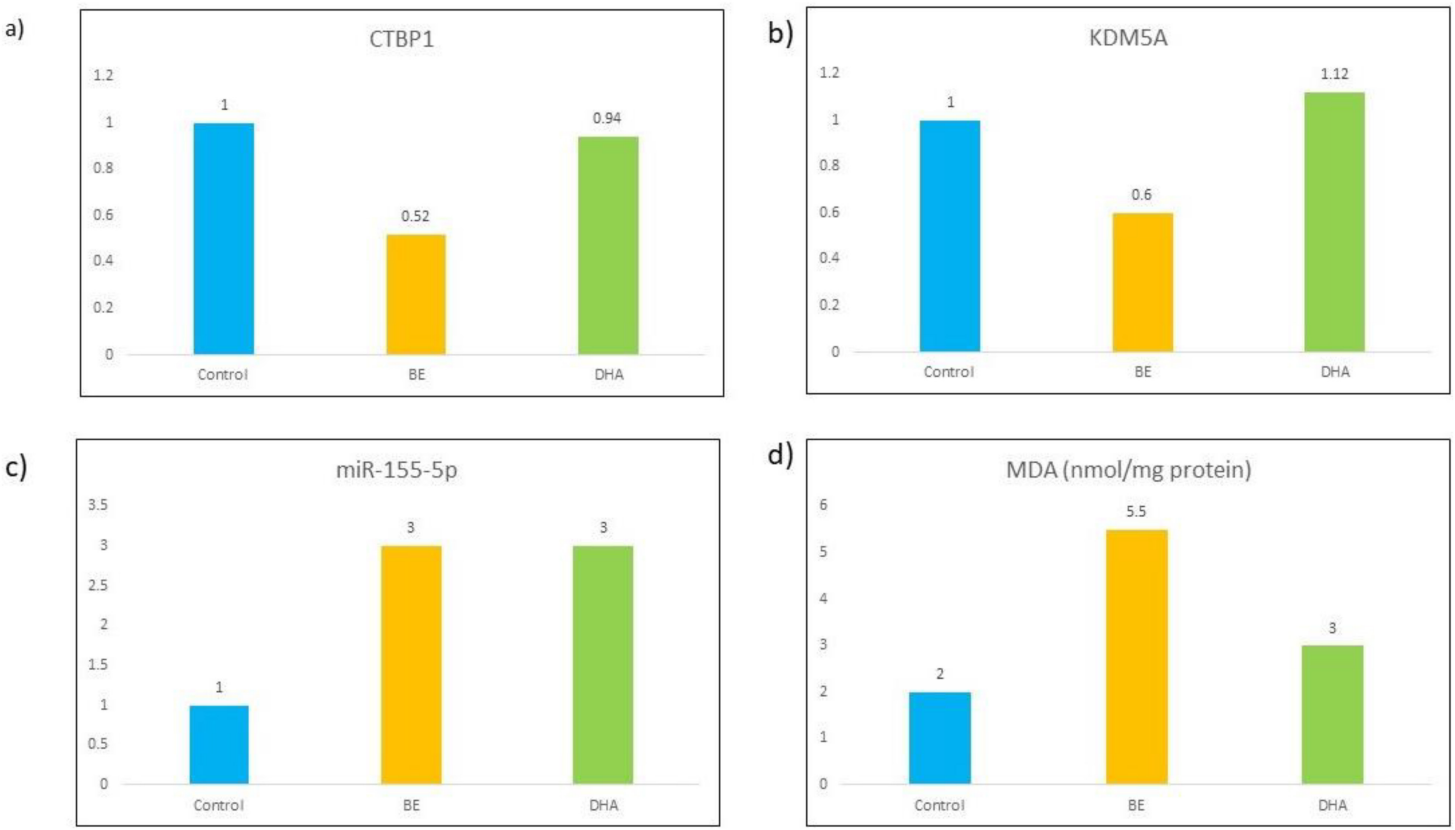
Relative expression of CTBP1 **(a)**, KDM5A **(b)**, miR-155-5p **(c)**, and MDA **(d)** in Control, BE, and BE + DHA groups (*in vivo* rat cortex). BE, bilirubin encephalopathy.

These results indicate that bilirubin exposure disrupted the CTBP1/miR-155-5p/KDM5A regulatory axis, whereas DHA treatment partially restored the expression of CTBP1 and KDM5A while suppressing miR-155-5p upregulation (Figure 7 and Table 5).

**TABLE 5:** Gene and protein expression (Relative Fold Change).

Group	miR-155-5p	CTBP1	KDM5A
Control	1.0 ± 0.0	1.0 ± 0.0	1.0 ± 0.0
BE	3.0 ± 0.5	0.52 ± 0.1	0.60 ± 0.08
BE + DHA	1.4 ± 0.3	0.94 ± 0.15	1.12 ± 0.12

### *In vitro* validation of miR-155-5p targeting of KDM5A

To verify whether miR-155-5p directly targets KDM5A, a luciferase reporter assay was performed in HEK293 cells. Transfection with a miR-155-5p mimic significantly reduced luciferase activity (∼40%) compared with controls (*p* < 0.01). In contrast, inhibition of miR-155-5p increased reporter activity, indicating reduced repression of the KDM5A 3′UTR.

Co-transfection with a CTBP1 overexpression plasmid partially restored luciferase activity, suggesting that CTBP1 modulates the miR-155-5p–KDM5A interaction.

**FIGURE 8:**
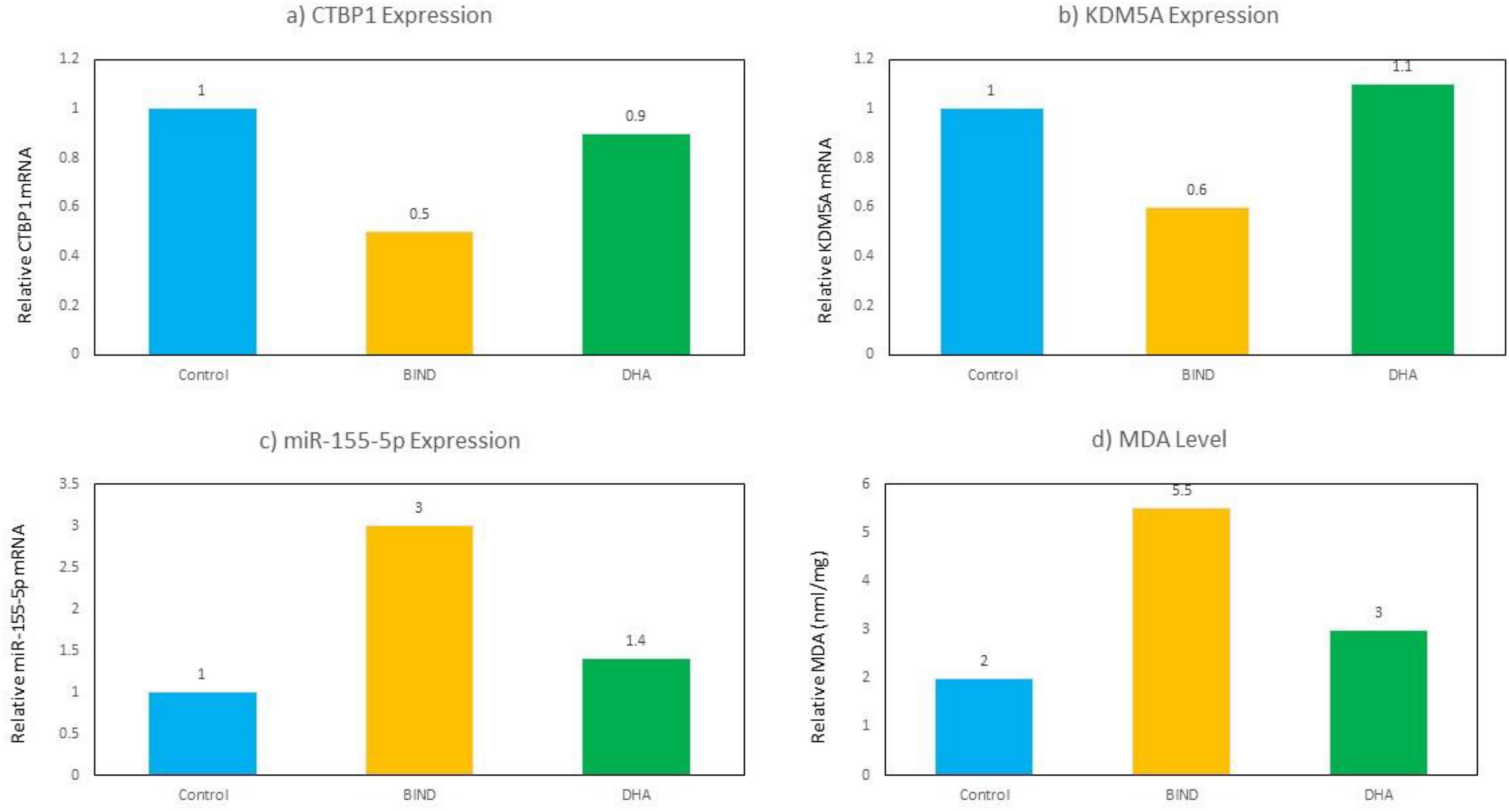
Luciferase reporter assay demonstrating the interaction between miR-155-5p and the KDM5A 3′UTR in HEK293 cells. **(a)** Control group, **(b)** miR-155-5p mimic, **(c)** miR-155-5p inhibitor, and **(d)** co-transfection with CTBP1 overexpression plasmid. Transfection with miR-155-5p mimic significantly reduced luciferase activity, while inhibition of miR-155-5p increased reporter activity. Co-transfection with CTBP1 partially restored luciferase activity.

Gene-expression changes are illustrated in Figure 7, while Table 5 provides the quantitative RT-qPCR values. The luciferase reporter assay results are shown in Figure 8, and Table 6 summarizes the overall behavioral, biochemical, and molecular outcomes across experimental groups.

**TABLE 6:** Behavioral, biochemical and molecular parameters.

Parameter	Control	BE	BE + DHA	*p*-value
Righting reflex (s)	3.18 ± 0.78	8.18 ± 1.97	4.16 ± 0.89	<0.0001
Geotaxis time (s)	5.82 ± 1.60	16.06 ± 5.23	7.88 ± 1.76	<0.0001
NSE (ng/mL)	5.05 ± 0.92	15.96 ± 2.77	7.09 ± 1.44	<0.0001
Brain Bilirubin (μg/g)	0.31 ± 0.09	8.22 ± 1.05	3.20 ± 0.84	<0.0001
MDA (nmol/mg)	1.99 ± 0.38	5.73 ± 0.58	2.99 ± 0.50	<0.0001
miR-155-5p (fold change)	1.00 ± 0.00	3.00 ± 0.50	1.40 ± 0.30	<0.0001
CTBP1 (fold change)	1.00 ± 0.00	0.52 ± 0.10	0.94 ± 0.15	<0.0001
KDM5A (fold change)	1.00 ± 0.00	0.63 ± 0.05	1.14 ± 0.11	<0.0001

Table 6 provides a comprehensive comparison of behavioral, biochemical, and molecular parameters among the study groups. The BE group exhibited significant impairments, including delayed reflexes (righting reflex: 8.18 ± 1.97 s), elevated neuronal injury markers (NSE: 15.96 ± 2.77 ng/mL), high brain bilirubin (8.22 ± 1.05 μg/g), oxidative stress (MDA: 5.73 ± 0.58 nmol/mg), and dysregulation of the CTBP1/miR-155-5p/KDM5A axis. DHA treatment markedly improved these parameters, restoring reflex performance, lowering NSE and MDA, reducing brain bilirubin, and normalizing gene expression profiles. All differences were statistically significant (*p* < 0.0001), confirming DHA’s robust neuroprotective effects in bilirubin-induced encephalopathy (Table 6).

Our results showed that DHA intervention substantially improved neurobehavioral outcomes and reduced neuropathology in neonatal rats with bilirubin-induced encephalopathy. These benefits were associated with: (1) enhanced clearance of bilirubin from serum and brain, (2) reduction of neuronal injury markers and apoptosis, (3) attenuation of oxidative stress and ferroptosis, and (4) modulation of the CTBP1–miR-155–KDM5A molecular pathway toward a neuroprotective profile. No significant differences were observed between the DHA-treated group and controls in many measures (e.g., by 72 h, DHA group’s bilirubin, ROS, CTBP1/KDM5A levels were statistically indistinguishable from controls), underscoring DHA’s efficacy in mitigating the damage.

## Discussion

The present study demonstrates that docosahexaenoic acid (DHA) attenuates multiple features of bilirubin-induced neurotoxicity in neonatal rats. DHA-treated animals exhibited improved neurobehavioral performance, lower serum and brain bilirubin levels at later time points, reduced neuronal injury markers, and decreased cortical apoptosis. In addition, DHA treatment was associated with modulation of oxidative stress markers and changes in the expression of the CTBP1/miR-155-5p/KDM5A signaling pathway. These findings suggest that DHA may mitigate several pathological processes associated with bilirubin-induced brain injury.

Our findings align with a growing body of BE research. Curcumin robustly protects the Gunn rat brain from bilirubin-induced injury, mitigating oxidative stress and behavioral deficits (17, 18). Proteomic and functional studies now implicate ferroptosis as a key death program in hyperbilirubinemia, nominating lipid-peroxidation control via the ACSL4/GPX4 axis as a therapeutic lever (7, 15, 16). Within this context, DHA’s antioxidant and membrane-stabilizing effects—and its potential to restrain ferroptosis—provide a mechanistic rationale for the benefits observed here (9, 17).

Regarding neurobehavioral outcomes; the untreated bilirubin-exposed rats showed delayed developmental reflexes (righting and geotaxis), reflecting sensorimotor impairment consistent with moderate kernicterus. DHA supplementation markedly improved these neurological outcomes, indicating better functional preservation of the central nervous system. This aligns with prior observations that DHA can enhance functional recovery in neonatal brain injury models. For instance, in a neonatal hypoxia-ischemia model, DHA-treated animals had better motor scores and reflexes compared to untreated ones. Similarly, our results mirror studies that found improved behavioral scores in bilirubin-exposed rats given ω-3 PUFAs (19). The fact that DHA nearly normalized the righting reflex by postnatal day 9 in our study is particularly encouraging; it suggests that DHA not only prevents neuronal death but also supports the maturation and integration of neural circuits necessary for reflex development. DHA’s role in facilitating synaptogenesis and neurite outgrowth might underlie these functional benefits, as it could help maintain neural connectivity despite bilirubin’s neurotoxic insult.

The lower bilirubin levels observed in DHA-treated animals may reflect several interacting factors. In addition to potential effects of DHA on oxidative stress and cellular responses, variability in bilirubin burden during experimental exposure may also depend on differences in phenotypic susceptibility and developmental maturation of hepatic bilirubin clearance mechanisms. In neonatal rodents, hepatic conjugation capacity and bilirubin transport systems continue to mature during the early postnatal period, which may influence circulating bilirubin concentrations and tissue accumulation. Experimental studies have shown that both the severity of bilirubin exposure and the developmental state of bilirubin metabolism pathways can affect the extent of brain bilirubin deposition and neurological injury.

Previous studies from our research group and others have primarily reported neuroprotective effects of DHA without a consistent reduction in circulating bilirubin levels. For example, the study by Hao et al. (9) demonstrated that DHA attenuated neuronal injury and improved neurological outcomes in bilirubin toxicity models, but did not observe a clear decrease in serum bilirubin concentrations. Similarly, clinical observations reported in subsequent work emphasized improvements in neurological status rather than direct bilirubin-lowering effects (8, 9, 20).

In contrast, the present study shows a more pronounced separation between BE and BE + DHA groups in the serum bilirubin time course. This difference may reflect variations in experimental design, bilirubin administration protocols, DHA dosing schedules, or analytical methods used for bilirubin measurement. Importantly, these findings should not be interpreted as establishing DHA as a primary bilirubin-lowering therapy. Instead, they suggest that DHA may indirectly influence bilirubin handling while primarily exerting neuroprotective effects through antioxidant, anti-apoptotic, and molecular regulatory mechanisms.

Thus, the current results extend previous work by demonstrating that DHA not only mitigates bilirubin-induced neuronal injury but may also modestly influence bilirubin dynamics under certain experimental conditions. Further studies will be required to determine whether this effect is reproducible and mechanistically significant (21, 22).

In the context of bilirubin encephalopathy, a more intact BBB would limit bilirubin entry into the brain. Our observation of less bilirubin pigment in DHA-treated brains is consistent with improved BBB function. Additionally, DHA’s incorporation into cell membranes might protect them from bilirubin’s detergent-like disruptive action. Improved systemic perfusion or cardiac output in healthier DHA-treated pups might also facilitate bilirubin distribution away from the brain. While further studies are needed to clarify the exact mechanism, the net effect is clearly beneficial lower brain bilirubin likely translates into less neuronal uptake of the toxin and hence reduced injury. Consistent with the observed biochemical and histological findings, DHA treatment was associated with reduced neuronal injury and apoptotic changes in cortical tissue. Neonatal neurons are highly vulnerable to bilirubin, undergoing apoptosis through mitochondrial pathways triggered by bilirubin-induced oxidative stress and calcium dysregulation (23).

DHA can intervene at multiple points: it is known to preserve mitochondrial integrity and function (24), and it can upregulate anti-apoptotic proteins (such as Bcl-2) while downregulating pro-apoptotic factors (like Bax and caspases) (25). Our histological data concur, showing reduced pyknosis and TUNEL staining with DHA. These findings are consistent with de Torres et al. (26), who reported that DHA-treated hyperbilirubinemic neonatal rats exhibited restoration of normal brain architecture and a marked decrease in cell death markers (26). Similarly, Zhu et al. (27) found that DHA administration after traumatic brain injury mitigated histopathological damage and apoptosis in the rat brain. The convergence of evidence from different models (bilirubin toxicity, hypoxia, trauma) suggests that DHA’s anti-apoptotic, neuroprotective effects are robust and generalizable (27).

Regarding oxidative stress and ferroptosis; a central novel aspect of our study is the demonstration that oxidative stress and ferroptosis are operative in BE and that DHA can significantly modulate these processes. We found elevated MDA and depressed GPX4 in the brains of jaundiced rats, which is characteristic of ferroptotic damage (28). This aligns with emerging understanding that bilirubin neurotoxicity involves not only excitotoxicity and apoptosis, but also oxidative lipid damage. Unconjugated bilirubin in excess can paradoxically act as a pro-oxidant, especially when the antioxidant reserves (like glutathione) are overwhelmed. The concomitant rise in PTGS2 (COX-2) we observed is notable PTGS2 is often used as a marker of ferroptosis in tissues, and its elevation in BE brains supports the involvement of ferroptosis. Furthermore, evidence from other studies shows that interventions which bolster antioxidant defenses or iron sequestration can attenuate bilirubin toxicity. For example, a recent study found that orexin-A alleviated post-traumatic ferroptosis in rat cortex by activating Nrf2/HO-1, restoring GPX4 and lowering PTGS2 (29).

Our results suggest DHA may act in an analogous manner indeed DHA has been reported to activate Nrf2 signaling in neural cells and to preserve GPX4 in models of oxidative injury. By integrating into neuronal membranes, DHA could also directly reduce lipid peroxidation propagation, given that DHA itself, when enzymatically oxygenated, yields neuroprotective mediators (like neuroprotectin D1) rather than toxic peroxides (30). The significant drop in MDA with DHA treatment (to near-control levels) in our study is a strong indicator that DHA effectively quenched the lipid peroxidation cascade initiated by bilirubin. In doing so, DHA likely interrupted the feedback loop of ferroptosis, wherein lipid ROS lead to further iron-dependent reactions and cell death. This is a novel therapeutic angle for bilirubin encephalopathy previously, treatments have not specifically targeted ferroptosis. Our data provide proof-of-concept that targeting ferroptotic pathways (whether via DHA or other antioxidants) holds promise in ameliorating bilirubin-induced neural injury, complementing the bilirubin-lowering strategies.

Regarding CTBP1/miR-155-5p/KDM5A pathway; one of the most significant contributions of this work is elucidating how DHA impacts the CTBP1–miR-155–KDM5A axis in the context of bilirubin neurotoxicity. This might be the first study to implicate this pathway in bilirubin-induced encephalopathy. We found that bilirubin exposure led to downregulation of CTBP1 and KDM5A, accompanied by a surge in miR-155-5p. MiR-155 is a well-known pro-inflammatory microRNA, often upregulated in microglia and macrophages during CNS injury (31). In neonatal bilirubin toxicity, elevated miR-155 could exacerbate neuroinflammation (for example, by suppressing negative regulators of inflammation such as SOCS1, as shown in other contexts) and also directly promote neuronal dysfunction. KDM5A, on the other hand, is a chromatin-modifying enzyme; while it has mostly been studied in cancer and stem cells, recent evidence suggests epigenetic regulation is crucial in neural injury responses. KDM5A can influence the expression of genes involved in cell survival and differentiation. A study by He et al. (32) showed that altering the CTBP1/miR-155/KDM5A loop affects cell survival pathways in neurons (32).

Our findings are in agreement with the study revealing the reduced CTBP1 and KDM5A likely contributed to the vulnerability of neurons in bilirubin encephalopathy, whereas DHA’s ability to restore their expression was associated with improved neuronal survival. How might DHA normalize this pathway? One possibility is that DHA’s activation of nuclear receptors (such as PPARs) or transcription factors indirectly upregulates CTBP1 expression, as CTBP1 is a transcriptional co-repressor that might be stabilized during anti-inflammatory responses. Additionally, DHA’s reduction of miR-155 could be through its immunomodulatory actions on microglia; less activated microglia release fewer exosomal miR-155 in the CNS. Interestingly, a lncRNA CTBP1-AS2 has been identified that sponges miR-155 in certain cell types; DHA might upregulate such protective lncRNAs in glial cells, thereby lowering miR-155 bioavailability. By whatever precise mechanism, the net effect observed was that DHA kept miR-155 in check. This is crucial because excessive miR-155 in the brain has been linked to neuroinflammation, synaptic dysfunction, and even autophagy-related neuronal death (33, 34).

Our study suggested that mitigating miR-155 overexpression (via CTBP1 upregulation) is a novel route through which DHA confers neuroprotection in bilirubin toxicity. Upregulation of KDM5A in DHA-treated brains may also have downstream benefits. KDM5A removes methyl groups from H3K4 on histones, generally repressing transcription of target genes. Paradoxically, in neural injury, some aberrant genes (e.g., those driving cell cycle re-entry in neurons or pro-death pathways) might need to be repressed for neurons to survive. It was recently shown that KDM5A helps maintain neural stem cells and prevents premature differentiation (35); in mature neurons, KDM5A might suppress genes that are deleterious when aberrantly expressed. Our results align with the concept that restoring KDM5A could reset the epigenetic state toward one favoring cell survival and repair. It is also conceivable that KDM5A participates in DNA damage response or chromatin remodeling after bilirubin-induced stress, aiding in recovery. The details of KDM5A’s role in neurons warrant further investigation, but our data firmly establish that DHA prevents the loss of KDM5A seen with bilirubin injury, which could be integral to its protective effect.

It is informative to compare DHA’s effects with other interventions reported for bilirubin encephalopathy. Recent study by Yan et al. (36) showed that a maternal diet rich in linoleic acid (omega-6 fatty acid from sunflower seed oil) improved outcomes in suckling. The mechanism there was attributed to activation of autophagy in neurons, which helped clear toxic protein aggregates induced by bilirubin (36). While linoleic acid and DHA are different classes of fatty acids, both highlight the potential of dietary lipids in modulating neurotoxic cascades. Autophagy activation (as seen with linoleic acid) and ferroptosis inhibition might be complementary; indeed, some studies suggest autophagy can either promote or inhibit ferroptosis depending on context. It would be interesting in the future to see if DHA also influences autophagy in bilirubin toxicity, for example, by affecting expression of autophagy-related genes or the turnover of damaged mitochondria (mitophagy). Another approach has been the use of antioxidant or anti-inflammatory agents. For instance, N-acetylcysteine (NAC), a glutathione precursor, has been tested in Gunn rat models and showed reduced oxidative brain damage. However, NAC’s ability to cross the BBB is limited. DHA, being highly lipophilic, is readily incorporated into the brain, making it an attractive long-term preventive supplement for at-risk infants. Curcumin and mesenchymal stem cell therapy have also been explored in experimental kernicterus, targeting inflammation and neurotrophic support, respectively (37, 38).

### Limitations

While our study provides important insights, several limitations warrant consideration. First, although our animal model is well established, it may not fully recapitulate the timing and complexity of human bilirubin encephalopathy, so translational caution is needed. Second, our experiments were limited to 72 h and did not assess long-term neurodevelopmental outcomes, phototherapy or exchange transfusion interactions, or alternate DHA dosing regimens. Third, our mechanistic findings on the CTBP1/miR-155-5p/KDM5A pathway are associative rather than causative and require validation with genetic or pharmacologic manipulation. Finally, our biochemical measures capture overall oxidative stress but not cell-type specificity. Despite these limitations, our findings offer a solid foundation for future studies and support the potential of DHA as an adjunctive therapy in neonatal bilirubin encephalopathy. Although both male and female pups were included, our study was not powered to evaluate sex differences; future studies will be needed to determine whether DHA’s effects vary by sex. Because our study did not include direct manipulation of CTBP1, miR-155-5p, or KDM5A, we cannot confirm a causal relationship. These findings should be interpreted as associations, and future studies using knockdown, overexpression, or pharmacologic modulation will be necessary to establish causality.

### Future directions

Future studies should clarify DHA’s effects on microglial activation and miRNA regulation (including miR-155, miR-122, and miR-125b) and test whether combining DHA with ferroptosis inhibitors yields additive neuroprotection. Translational work using preterm or genetically jaundiced models, as well as evaluating brain-targeted DHA formulations such as lysophosphatidylcholine, could further optimize its therapeutic potential. Collectively, these investigations will refine DHA-based strategies for preventing neonatal bilirubin encephalopathy.

### Clinical implications

Our findings suggest that DHA, already used as a nutritional supplement in premature infants, could be repurposed as an adjunct neuroprotective therapy for neonates at risk of bilirubin-induced brain injury. By modulating the CTBP1/miR-155-5p/KDM5A pathway and reducing oxidative stress, DHA may promote cortical repair and attenuate progression to acute bilirubin encephalopathy. Given its established safety profile, enteral DHA administration alongside conventional treatments warrants clinical investigation to improve neurodevelopmental outcomes and reduce kernicterus-related disabilities.

## Conclusion

This study shows that docosahexaenoic acid (DHA) significantly mitigates neuropathological and functional deficits in a rodent model of bilirubin-induced encephalopathy. DHA improved neurobehavioral outcomes, reduced neuronal apoptosis, and preserved cortical structure while normalizing the CTBP1/miR-155-5p/KDM5A axis and suppressing oxidative stress and ferroptosis. These findings highlight DHA’s multifaceted neuroprotective mechanisms and support its potential as a therapeutic adjunct for neonates with severe hyperbilirubinemia.

## Data Availability

The original contributions presented in the study are included in the article/Supplementary material, further inquiries can be directed to the corresponding author.
