# Supplementary figures and images for: Docosahexaenoic acid attenuates neuropathological alterations in a rodent model of neonatal bilirubin-induced encephalopathy
Source: Front Med (Lausanne). 2026 Apr 2;13:1633947. doi: 10.3389/fmed.2026.1633947 (PMC13083093; doi:10.3389/fmed.2026.1633947)

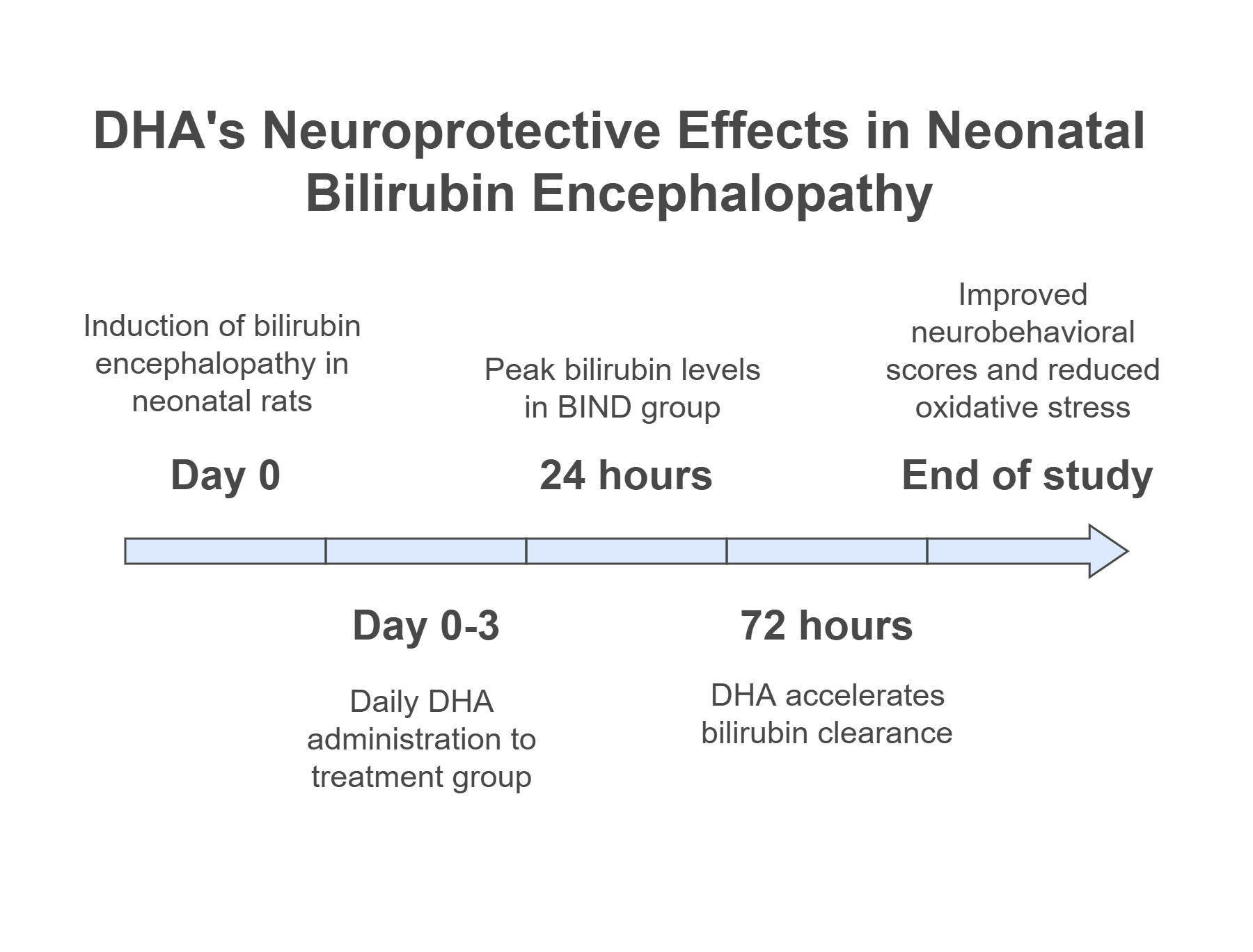

Supplement: Supplementary file 1 [file Image_1.png]
